# Meta-learning approach for bacteria classification and identification of informative genes of the *Bacillus megaterium*: tomato roots tissue interaction

**DOI:** 10.1007/s13205-023-03690-0

**Published:** 2023-07-11

**Authors:** Vânia Rodrigues, Sérgio Deusdado

**Affiliations:** 1 USAL—Universidad de Salamanca, 37008 Salamanca, Spain; 2 Instituto Politécnico de Bragança, CIMO—Centro de Investigação de Montanha, 5301-855 Bragança, Portugal

**Keywords:** Machine learning, Meta learners, Informative genes, *Solanum lycopersicum*, *Bacillus megaterium*

## Abstract

Plant growth-promoting rhizobacteria (PGPRs) are bacteria that colonize the plant roots. These beneficial bacteria have an influence on plant development through multiple mechanisms, such as nutrient availability, alleviating biotic and abiotic stress, and secrete phytohormones. Therefore, their inoculation constitutes a powerful tool towards sustainable agriculture and crop production. To understand plant-PGPRs interaction we present the classification of PGPR using machine learning and meta-learning classifiers namely Support Vector Machine (SVM), Kernel Logistic Regression (KLR), meta-SVM and meta-KLR to predict the presence of *Bacillus megaterium* inoculated in tomato root tissues using publicly available transcriptomic data. The original dataset presents 36 significantly differentially expressed genes. As the meta-KLR achieved near-optimal performance considering all the relevant metrics, this meta learner was afterwards used to identify the informative genes (IGs). The outcomes showed 157 IGs, being present all significantly differentially expressed genes previously identified. Among the IGs, 113 were identified as tomato genes, 5 as *Bacillus subtilis* proteins, 1 as *Escherichia coli* protein and 6 were unidentified. Then, a functional enrichment analysis of the tomato IGs showed 175 biological processes, 22 molecular functions and 20 KEGG pathways involved in *B. megaterium–*tomato interaction. Furthermore, the biological networks study of their *Arabidopsis thaliana* orthologous genes identified the co-expression, predicted interaction, shared protein domains and co-localization networks.

**Supplementary Information:**

The online version contains supplementary material available at 10.1007/s13205-023-03690-0.

## Introduction

Plants are colonized by complex bacterial communities that play different roles in plant health and growth (Brader et al. 2017). Some bacteria are pathogenic and cause diseases, and others can promote plant growth by enhancing the tolerance to biotic and abiotic stresses as well as the nutrient acquisition, but a large fraction of these bacteria have no known function to their host (Bulgarelli et al. 2013). Tomato (*Solanum lycopersicum* L.) is an excellent model for studying plant–microbe interactions, for basic and applied research on fruit quality and other physiological traits (Bai et al. 2018; Karlova et al. 2014), but a comparison of its associated bacterial community is still incipient (Dong et al. 2019; Yang et al. 2022). Characterization of bacterial communities associated with tomato plants will contribute to identifying potential candidates for biological control (Romero et al. 2016). Several studies have pointed the influence of *Bacillus megaterium* in plant growth promotion (Akinrinlola et al. 2018; López-Bucio et al. 2007; Ortíz-Castro et al. 2008). In tomato seedlings using *Arthrobacter* and *B. megaterium* isolated from the rhizosphere of wild plants grown on saline–alkaline alleviated salt stress (Fan et al. 2016). Understanding the mechanisms involved in the link among specific microbes, the ecosystem properties, and the plant under study, is an ongoing challenge due to the complexity of the different biological systems. To assist this challenge, the integration of advances in DNA/RNA sequencing technology with machine learning (ML) can yield relevant information.

ML methods are widely used in several research fields, such as climate change and human health, clinical decision-making, plant science (van Dijk et al. 2021), and in the field of microbiology (Peiffer-Smadja et al. 2020). ML addresses the analysis of large amounts of data by discovering relationships between the target output and the input attributes, and it is mostly used for prediction and classification tasks. The analysis of metagenomic microbial bring several difficulties (Taş et al. 2021). Data are typically high dimensional with a small number of samples collected in each study. Furthermore, sequencing results are noisy and yield sparse datasets (Li 2015). This kind of dimensionality has critical importance to conduct feasible and pilot work, however, it can lead to biased ML performance estimates. The ML validation process should be designed to help avoid optimistic performance estimates. Commonly, the higher the ratio of attributes to sample sizes the more likely that a ML model will fit the noise in the data instead of the underlying pattern (Raudys and Jain 1991). To aid in this, meta-learning algorithms (Vanschoren 2019) of attribute-selected classifiers implement an evaluator to reduce the dimension of training and test data before being passed on to a classifier (Witten and Frank 2005), improving its performance.

This study aims to discover a more complete characterization of the *B megaterium*—tomato roots tissue interaction based on a novel methodology integrating machine learning to the interpretation of the gene expression data generated in that interaction. To achieve that goal were applied ML classifiers and meta-learning algorithms of attribute-selected classifiers, developing into a model capable to predict the presence of *B. megaterium* inoculated in tomato root tissue and then used to identify potential informative genes related to their interaction under well-watered conditions. Based on its performance, Support Vector Machine (SVM) (Keerthi et al. 2001) and Kernel Logistic Regression (KLR) (Wahba et al. 1995) classifiers were selected. As SVM makes no assumption regarding the underlying data distribution, this may be advantageous for a small sample set. Additionally, it presents the ability to find a balance between accuracy and generalization. SVM can achieve good prediction accuracy for new observations despite large numbers of input variables. It is frequently used in genome-wide analysis and brain imaging, two application domains that often have small sample sizes but a very high number of attributes (Bzdok et al. 2018). The KLR model is a statistical classifier (Wahba et al. 1995) that generates a fit model by minimizing the negative log-likelihood with a quadratic penalty using the Broyden–Fletcher–Goldfarb–Shanno (BFGS) optimization (Smith et al. 2015). For each meta learner, meta-SVM and meta-KLR, the chi-squared statistic was applied as attribute evaluator. Then, functional enrichment analysis of gene ontology (GO) terms of the biological processes was carried out, as well as molecular functions and Kyoto Encyclopedia of Genes and Genomes (KEGG) analysis for the highest-rated genes of *S. lycopersicum* identified. Biological networks were also composed based on the corresponding *Arabidopsis thaliana* orthologous genes from heterogeneous data sources. The resulting networks can include physical interactions, co-expression, predicted interactions, shared protein domains, genetic interaction, and co-localization.

## Materials and methods

### Dataset collection

The original datasets were obtained from the GEO/NCBI database with reference accession: GSE106317. The used dataset was achieved by selecting the appropriate data for this work, specifically three replicates of root tomato wild-type cv. Pearson line non-inoculated under well-watered conditions (GSM2835866, GSM2835867, GSM2835868), and three replicates of *B. megaterium* inoculated root tomato wild-type cv. Pearson line under well-watered conditions (GSM2835869, GSM2835870, GSM2835871).

### Experimental procedures

The original datasets from GEO/NCBI database are recorded by log_2_ GC-RMA signals. Before applying the ML classifiers and meta-learning algorithms of attribute-selected classifiers, the inverse signals were obtained to achieve a more approximate behavior of the relative gene expression, and then they were normalized to [0, 1] on the assembled dataset. SVM and KLR classifiers were executed on the assembled dataset with different parameterization (see Section “Classifiers parameters tuning”).

To build the meta learners meta-SVM and meta-KLR, the chi-squared attribute evaluators were applied to reduce dimensionality before being passed on to SVM and KLR classifiers, respectively. The number of attributes to retain was chosen after several tests and validating the results of performance evaluation through comparison with results obtained when the classifier was applied to the original number of attributes. Three meta learners emerged as the best performers, meta-SVM-a (128 attributes), meta-SVM-b (99 attributes) and meta-KLR-b (700 attributes). Meta-KLR-a (700 attributes) is also presented to show the influence of *λ* parameter on the number of attributes retained. The metrics used for performance evaluation were accuracy, kappa coefficient, mean absolute error, precision and recall (Table 1). These experiments ran 10 times several schemes using leave-one-out cross-validation (LOOCV) testing with Paired T-Tester. Then, the meta learner with the best performance evaluation was used to identify IGs. To do this, the meta learner was used for estimating the accuracy and root mean squared error (RMSE) of attribute combinations subsets with 4 folds, using a threefold CV. If the standard deviation of the means exceeds 0.01, the attribute combinations are repeated, otherwise, the ranks were obtained according to the evaluation.

**Table 1:** Metrics of performance evaluation

Accuracy (ACC)	(TP + TN)/(TP + FP + FN + TN)
Kappa coefficient (κ)*	(Observed accuracy-Expected accuracy)/(1-Expected accuracy)
Mean absolute error (MAE)	$\left({\sum }_{i=1}^{n}|\mathrm{predicted i}-\mathrm{actual i}|\right)/(\mathrm{Total} \mathrm{predictions})$
Root mean squared error (RMSE)	sqrt(sum((x_i_-y_i_)^2^)/n)
Precision (PRE)	TP/(TP + FP)
Recall (REC)	TP/(TP + FN)

*TP* true positive, *TN* true negative, *FP* false positive, *FN* false negative, *x*_*i*_ vector of calculated values, *y*_*i*_ vector of actual values an *n* number of observations

*Kappa coefficient is interpreted using the guidelines outlined by Landis and Koch (1977), where the strength of the κ is interpreted in the flowing manner: 0.01–0.20 slight; 0.21–0.40 fair; 0.41–0.60 moderate; 0.61–0.80 substantial; 0.81–1.00 is almost perfect (Landis and Koch 1977)

The experimental work was based on WEKA, version 3.9.5, a data mining workbench publicly accessible at: www.cs.waikato.ac.nz/ml/weka/.

The Tomato Functional Genomics Database (TFGD) (Fei et al. 2011) was used to identify the gene ID of the probe ID from the Affymetrix genome array. Subsequently, protein sequences from tomatoes were aligned using NCBI BLAST (Altschul et al. 1990) BLASTP, against *A. thaliana* setting nonredundant protein sequences database using default parameters.

Utilizing g:Profiler (Raudvere et al. 2019), a functional enrichment analysis by GO terms of biological processes and molecular functions were performed to provide biological and molecular gene annotations of the highest-rated tomato genes. KEGG pathways analysis was also performed to understand their functions and interactions.

Resorting to GeneMANIA Cytoscape database (Montojo et al. 2010), the corresponding genes of the protein found by BLASTP with higher hit values (query coverage ≥ 90%, percent identity > 80% and e-value ≤ 0), were used to identify biological networks. Moreover, biological interpretation for the highest-rated genes set (IGs) were also based on literature.

To obtain the significantly differentially expressed genes (DEGs) (Benjamini & Hochberg false discovery rate) limma precision weights (vooma function) and quantile normalization with adjusted *p*-value < 0.05 were applied to the sample groups.

These sets of experiments were conducted on a computer with an Intel Core i7-5500U CPU 2.40 GHz processor, with 8 GB RAM.

### Classifiers parameters tuning

A grid search approach was used to optimize the parameters of SVM and KLR classifiers. The parameters optimized for SVM were regularization parameter *C* and polynomial kernel function *p*. The regularization parameter *C* weights the importance of misclassification and allows SVM to fit a linear separating hyperplane with some of the examples being misclassified. The Kernel function enables the separation of classes which are non-linearly separable in the original space with a linear hyperplane in a higher dimensional space. After performing a grid search, two SVM classifiers emerged as the best performers: SVM-a (*C* = 1 and *p* = 0.5); and SVM-b (*C* = 0.5 and *p* = 0.5). In KLR, the support vector parameters *λ* with different types of kernel functions were optimized. After performing a grid search, two KLR classifiers using a linear kernel function were revealed as the best performers, KLR-a (*λ* = 0.01) and KLR-b (*λ* = 0.001). All grid searches were executed using LOOCV.

## Results and discussion

The experimental work was carried out with the classifier’s parameterization and the meta-learners described in the previous section. SVM and KLR classifiers achieved similar performance evaluation, but affected by significant standard deviation (Table 2).

**Table 2:** SVM and KLR performance evaluation using LOOCV

	SVM-a	SVM-b	KLR-a	KLR-b
ACC (%) (st.dev.)	83.33 (37.58)	83.33 (37.58)	83.33 (37.58)	83.33 (37.58)
K (st.dev.)	0.83 (0.38)	0.83 (0.38)	0.83 (0.38)	0.83 (0.38)
MAE (st.dev.)	0.17(0.38)	0.17(0.38)	0.13 (0.22)	0.12 (0.23)
PRE (st.dev.)	1	1	1	1
REC (st.dev.)	0.67 (0.48)	0.67 (0.48)	0.67 (0.48)	0.67 (0.48)

SVM-a: C = 1 and p = 0.5, SVM-b: C = 0.5 and p = 0.5 KLR-a: λ = 0.01, KLR-b: λ = 0.001

Due to the small number of samples and higher number of attributes, meta learners were executed to reduce the dimensionality of the training and test data by applying chi-squared as attribute selection before being passed on to SVM-a, SVM-b, KLR-a, and KLR-b. Table 3 shows the performance evaluation of the meta learners arising from the previous classifiers.

**Table 3:** Meta-SVM and meta-KLR performance evaluation using LOOCV

	Meta-SVM-a^§1^	Meta-SVM-b^§2^	Meta-KLR-a ^§3^	Meta-KLR-b ^§3^
ACC (%) (st.dev.)	83.33 (37.58)	83.33 (37.58)	66.67 (47.54)	100
k (st.dev.)	0.83 (0.38)	0.83 (0.38)	0.67 (0.48)	1
MAE (st.dev.)	0.17 (0.38)	0.17 (0.38)	0.36 (0.32)*	0.10 (0.18)
PRE (st.dev.)	1	1	1	1
REC (st.dev.)	0.67 (0.48)	0.67 (0.48)	0.67 (0.48)	1

^§1^128 ranked attributes

^§2^99 ranked attributes and

^§3^700 ranked attributes

*Statistically different at significance level 0.05

The performance results of meta learners presented in Table 3 were achieved after having found the reduced number of attributes without affecting the performance evaluation of the used classifiers (Table 2), except for meta-KLR-a which was verified that with the same number of attributes used in meta-KLR-b, the performance was worse than with the original data size.

**Fig. 1:**
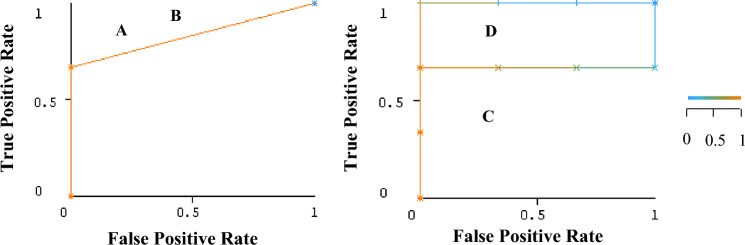
**A** meta-SVM-a: AUROC = 0.83, **B** meta-SVM-b: AUROC = 0.83, **C** meta-KLR-a: AUROC = 0.67, **D** meta-KLR-b: AUROC = 1

Overall, the reduction in the number of attributes resulted in the same performance of the SVM. For KLR, the performance improves when the complexity parameter λ is decreased. Other metric used was the area under ROC (AUROC), which measures the overall quality of a classifier (Fig. 1). Meta-KLR-b achieved AUROC = 1 (Fig. 1D), which means that positive and negative samples are correctly classified, whereas meta-SVM-a and meta-SVM-b achieved the same AUROC = 0.83. The meta-KLR-a showed the lowest AUROC = 0.67.

**Fig. 2:**
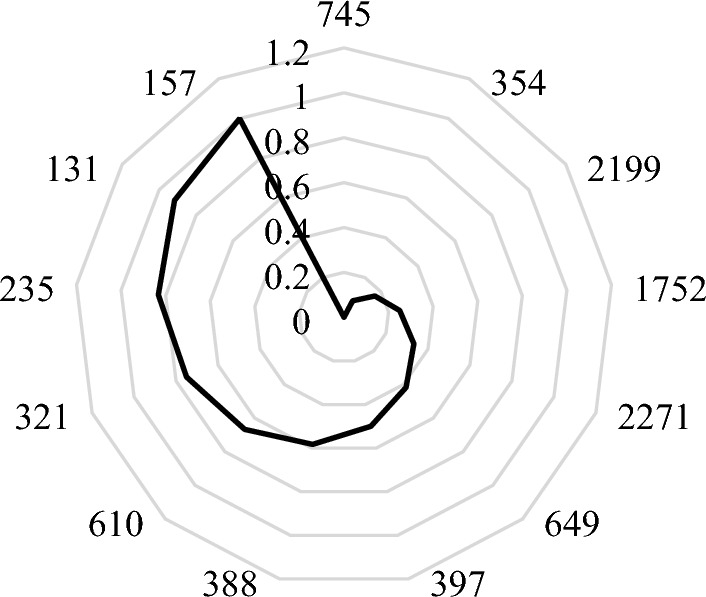
Number of attributes with its average merit using meta-KLR-b

Due to the results of the meta-KLR-b performance, it was afterwards used as an attribute evaluator, evaluating the worth of an attribute by estimation of the accuracy and root mean squared error of attribute combinations subsets with 4 folds, using a threefold CV. Subsequently, the attributes were ranked according to the average merit (Fig. 2). This meta learner gathered the genes into 13 groups. The four highest scores are: 157 genes with a score of 1, 131 genes with a score of 0.917 ± 0.118, 235 genes with a score of 0.833 ± 0.118 and 321 genes with a score of 0.75 ± 0.204.

The gene IDs of the 157 attributes identified with a score of 1 were searched in Tomato Functional Genomics Database (TFGD) and compared to the references reported by the authors of the gene expression dataset (Ibort et al. 2018). There were also present two probes IDs identified as 1 gene of *Escherichia coli*, 36 probes IDs identified as 5 genes of *Bacillus subtilis* and 6 were unidentified.

In the assembled dataset were found 36 significantly differentially expressed genes (DEGs) with adjusted *p*-value < 0.05. These DEGs are captured by 157 IGs identified by meta-KLR-b. It is important to mention that the information from DEGs was not used and still the meta-learning approach identified them as part of the IGs.

### Highest score attributes (IGs)

Through the meta-KLR-b, 157 attributes were identified with a score of 1. Among these, 113 were identified as tomato genes (Appendix A). Approximately 20% of them are reported to have a role in plant development and growth. A recent study suggests RPL8 (Les.3790.1.S1_at) gene as an optimal internal control to analyze gene expression in developing fruits. This gene was identified as an internal control of dwarf tomato cultivar (Choi et al. 2018). There is a study that suggests that RP genes are useful markers to monitor growth states for microarray analysis (Tatematsu et al. 2008). The gene calcineurin subunit B (LOC101250979, LesAffx.69816.1.S1_at), regulates growth in *Neurospora crassa* (Tamuli et al. 2016). In the fungal kingdom, calcineurin is responsible for maintaining a diverse range of cellular processes such as, growth, morphogenesis, stress response, ion homeostasis and pathogenicity by activating downstream events (Park et al. 2019).

According to Uniprot, probable histone-lysine N-methyltransferase ATXR5 (LOC101244542, LesAffx.67341.1.S1_at) gene has the molecular functions of both metal ion and histone binding and in transcription regulator activity. Exhibiting as biological processes the negative regulation of transcription, DNA-templated, positive regulation of transcription by RNA polymerase II. K( +) efflux antiporter 2, chloroplastic gene (LOC101246664, LesAffx.17169.1.A1_at) is involved in potassium:proton antiporter activity as molecular function. Acetolactate synthase 2, chloroplastic-like (LOC101254112, Les.463.1.S1_at) gene is involved in acetolactate synthase activity, flavin adenine dinucleotide binding, magnesium ion binding and thiamine pyrophosphate binding as molecular functions. Presenting isoleucine biosynthetic process, response to herbicide and valine biosynthetic process as biological processes. A recent study suggests that acetolactate synthase is involved in volatile organic compounds (VOCs) production/degradation by the acetoin biosynthesis pathway (Nascimento et al. 2020).

Probable 6-phosphogluconolactonase 4, chloroplastic (LOC101244165, Les.4800.1.S1_at) gene is involved in carbohydrate degradation through the pentose phosphate pathway and the urease accessory protein F (Uref, Les.4305.1.S1_at) gene also identified, is involved in nitrogen, sulfur and phosphorus metabolism through urea degradation and transport (Nascimento et al. 2020). Peroxisome biogenesis protein 19-2 (LOC101252997, Les.4229.1.S1_at) was also identified. According to Uniprot, this gene presents as a molecular function the peroxisome membrane targeting sequence binding and as a biological process the protein import into the peroxisome membrane. In plants, peroxisome plays multiple roles, including metabolism, development, and stress response. Defective peroxisome biogenesis and development retard adaption, plant growth, and reproduction (Jia et al. 2017).

According to Uniprot, Hop-interacting protein THI037 (LOC101055522, LesAffx.68102.1.A1_at) gene is involved in two molecular functions, metal ion binding and ubiquitin-protein ligase activity. Cellulose synthase-like protein G2 (LOC101255510, Les.2316.2.A1_at) gene was also identified. It is involved in the cellulose biosynthetic process, cell wall organization and plant-type primary cell wall biogenesis in *A. thaliana*. Heat stress transcription factor A-6b (LOC101257776, LesAffx.56.3.S1_at) gene is involved in the cellular response to heat and in regulation of transcription by RNA polymerase II. This heat stress factor connects ABA signaling and ABA-mediated heat responses (Huang et al. 2016).

3-hydroxy-3-methylglutaryl-coenzyme A reductase 1 (HMGR, Les.4372.1.S1_at) gene catalyzes the synthesis of mevalonate, the specific precursor of all isoprenoid compounds present in plants. According to Uniprot, this gene is involved in one molecular function, hydroxymethylglutaryl-CoA reductase (NADPH) activity and three biological processes, coenzyme A metabolic process, isoprenoid biosynthetic process and sterol biosynthetic process. Calcium load-activated calcium channel (LOC101257715, Les.3036.2.S1_at) gene is related to the calcium-selective channel required to prevent calcium stores from overfilling, having as biological processes the cellular calcium ion homeostasis and endoplasmic reticulum calcium ion homeostasis. Peroxidase 3 (LOC101244376, LesAffx.6103.1.S1_at) gene was also identified. Peroxidases are particularly abundant in root meristems and are involved in the formation and interconversion of reactive oxygen species (ROS), which play a critical role in root and root hair development (Díaz-Tielas et al. 2012). Pre-mRNA-splicing factor prp12 (LOC101261183, Les.3829.1.S1_at) gene was also identified. It is involved in mRNA splicing, via spliceosome (Gaudet et al. 2011).

Our results also infer that *B. megaterium* probably has an influence on the gene which regulates the nucleotide sugar transporters (NSTs). These transporters regulate glycosylation in plants. Although our knowledge on plant complex N-glycan functions is limited, genetic studies revealed the importance of complex N-glycans in the cellulose biosynthesis and growth under stress (Nagashima et al. 2018). An important function of NSTs is to supply substrates for glycosyltransferases in the synthesis of wall matrix polysaccharides and glycoproteins (Zhang et al. 2011). According to Uniprot database, the cytochrome b-c1 complex subunit 8 (LOC101267820, LesAffx.66052.1.S1_at) gene identified is a component of the mitochondrial respiratory chain. Cytochrome b-c1 complex have also been associated with the generation of reactive oxygen species (ROS) (Xia et al. 2013). ROS act as plant signaling molecules that participate in various processes such as growth and development (Choudhary et al. 2019). Malate dehydrogenase (LOC101253131, Les.3378.2.S1_at) gene was also identified. According to the TFGD, its biological processes include glycolysis, oxidation–reduction, malate metabolic process, tricarboxylic acid cycle, response to cadmium ion, carbohydrate metabolic process and response to salt stress. Calmodulin 1 (CaM1, Les.2645.2.S1_at) gene encodes a member of the calmodulin gene family that acts as a positive regulator of defense against wounding and *Botrytis cinerea* infections in tomato fruits (Peng et al. 2014). A recent study reported that calmodulin had broad expression patterns in abiotic stress conditions and with hormone treatments, in different tissues of stress-tolerant wild tomato species, *Solanum pennellii* (Shi and Du 2020). CaM gene interacts with a wide range of downstream target molecules that mediate diverse cell process (Bergey et al. 2014). A target protein known to bind CaM and mediate key plant cell process is mitogen-activated protein kinases (MAPKs). MAPK cascades are known to regulate processes involved in plants such as growth and development (Taj et al. 2010). According to Uniprot database, indole-3-glycerol-phosphate synthase, chloroplastic (LOC101250390, LesAffx.28368.1.S1_at) gene is involved in the tryptophan biosynthetic process. According to the TFGD, it is also involved in other biological processes such as metabolic, amino acid biosynthetic, tryptophan metabolic and aromatic amino acid family biosynthetic. Indole-3-glycerol-phosphate synthase gene is a branchpoint of indole-3-acetic acid biosynthesis from the tryptophan biosynthetic pathway in *A. thaliana*. Where this phytohormone plays a vital role in plant growth and development as a regulator of several biological processes (Ouyang et al. 2000). According to the TFGD, glutathione S-transferase U17 (LOC101250402, LesAffx.56167.1.S1_at) gene has as molecular function the glutathione binding, lyase activity, lactoylglutathione lyase activity and transferase activity. It presents as biological processes the auxin-mediated signaling pathway, response to stress, toxin catabolic process and in response to oxidative stress.

DNA (cytosine-5)-methyltransferase 1 (MET1, Les.94.1.S1_at) gene was also identified as IGs. A recent study (Yang et al. 2019) indicates that this gene also known as SIMET1, is required for maintaining a normal transcriptome and normal development of tomatoes. According to the TFGD, it is involved in several biological processes such as S-adenosylmethionine and S-adenosylhomocysteine metabolic processes, regulation of transcription, DNA-dependent, regulation of cell proliferation, maintenance of DNA methylation, regulation of gene expression and genetic imprinting. Probable serine/threonine protein kinase IREH1 (LOC101246150, Les.5407.1.S1_at) gene is involved in spermatid differentiation, cell communication, protein amino acid phosphorylation and in regulation of interleukin-12 biosynthetic process. Serine/threonine-protein kinase BSK2 (LOC101251509, Les.2487.1.A1_at) gene is involved in protein amino acid phosphorylation and glycolysis. SPY protein (spy, Les.3463.1.S1_at) gene is involved in several biological processes such as regulation of oxygen and reactive oxygen species metabolic process, gibberellic acid mediated signaling, flower development, multicellular organismal development and cytokinin mediated signaling. According to the TFGD, ethylene-inducible CTR1-like protein kinase (CTR1, Les.31.1.S1_s_at) gene is involved in various biological processes such as ethylene-mediated signaling pathway, actin cytoskeleton organization, protein amino acid phosphorylation, sugar-mediated signaling, gibberellin biosynthetic process, regulation of timing of transition from vegetative to reproductive phase, chemotaxis, and phagocytosis. This gene is involved in several molecular functions such as metal ion binding, protein tyrosine kinase activity, MAP kinase kinase kinase activity and magnesium ion binding.

According to the TFGD, the shaggy-related protein kinase kappa (LOC101265073, Les.4613.1.S1_at) gene is involved in several biological processes as hyperosmotic response, response to salt stress, protein amino acid phosphorylation and meristem structural organization. In regard to molecular functions this gene is involved in protein serine/threonine kinase activity, nucleotide binding and ATP binding. Non-specific lipid-transfer protein 2 (LE16, Les.1389.1.S1_at) gene is involved in transport, defense response, lipid transport, response to stress and response to the biotic stimulus. Elongation factor 1-beta 2 (LOC101268350, Les.2920.2.S1_at) gene is involved in defense response to bacterium and translational elongation. According to Uniprot, actin-related protein 3 (LOC101252768, Les.4758.1.S1_at) gene is involved in Arp2/3 complex-mediated actin nucleation (Gaudet et al. 2011). According to the TFGD, this gene is involved in actin filament organization, cell morphogenesis, response to stress, multicellular organismal development and regulation of actin filament polymerization. Regarding molecular functions, this gene presents ATP binding, protein binding, nucleotide binding and actin binding.

There are attributes assigned to *B. subtilis subsp. subtilis* str. 168 proteins, such as threonine synthase, and GTPase obgE involved in ribosome biogenesis and sporulation resulting in the formation of a cellular spore, lysA involved in the lysine biosynthetic process via diaminopimelate, polyA polymerase, and the anthranilate phosphoribosyltransferase is involved in the tryptophan biosynthesis (Gaudet et al. 2011). Another bacteria identified was *E. coli* through biotin synthetase.

### Functional enrichment analysis

To the best of our knowledge, g:Profiler is the reference web server for functional analysis that presents information on the Gene Ontology and the biological pathways involved in *S. lycopersicum*. It is important to refer that g:Profiler results are constantly updated, and the order of the identified gene set affects the results. From the 113 *S. lycopersicum* genes, 98 were reported, but two of them were not included in the survey due to ambiguous queries.

#### Biological process

The outcome showed that from the 96 tomato genes included in the survey, 63 were classified into 175 GO biological processes, as presented in Appendix B. More general the cellular and metabolic process are the processes with the highest number of genes assigned, as well as nitrogen compound metabolic process, which is one of the most important essential elements in plants, act as a limiting factor for crop yield and plant growth (Baslam et al. 2020), whereas more specific biological processes like nucleotide-sugar biosynthetic process, the lysine biosynthetic process, regulation of gibberellic acid mediated signaling pathway, DNA methylation on cytosine within a CG sequence, regulation of reactive oxygen species metabolic process, tryptophan biosynthetic process (Yang et al. 2022), calcium-mediated signaling are some of the biological processes with an important role in plant growth and development, although having less genes involved.

#### Molecular function

**Fig. 3:**
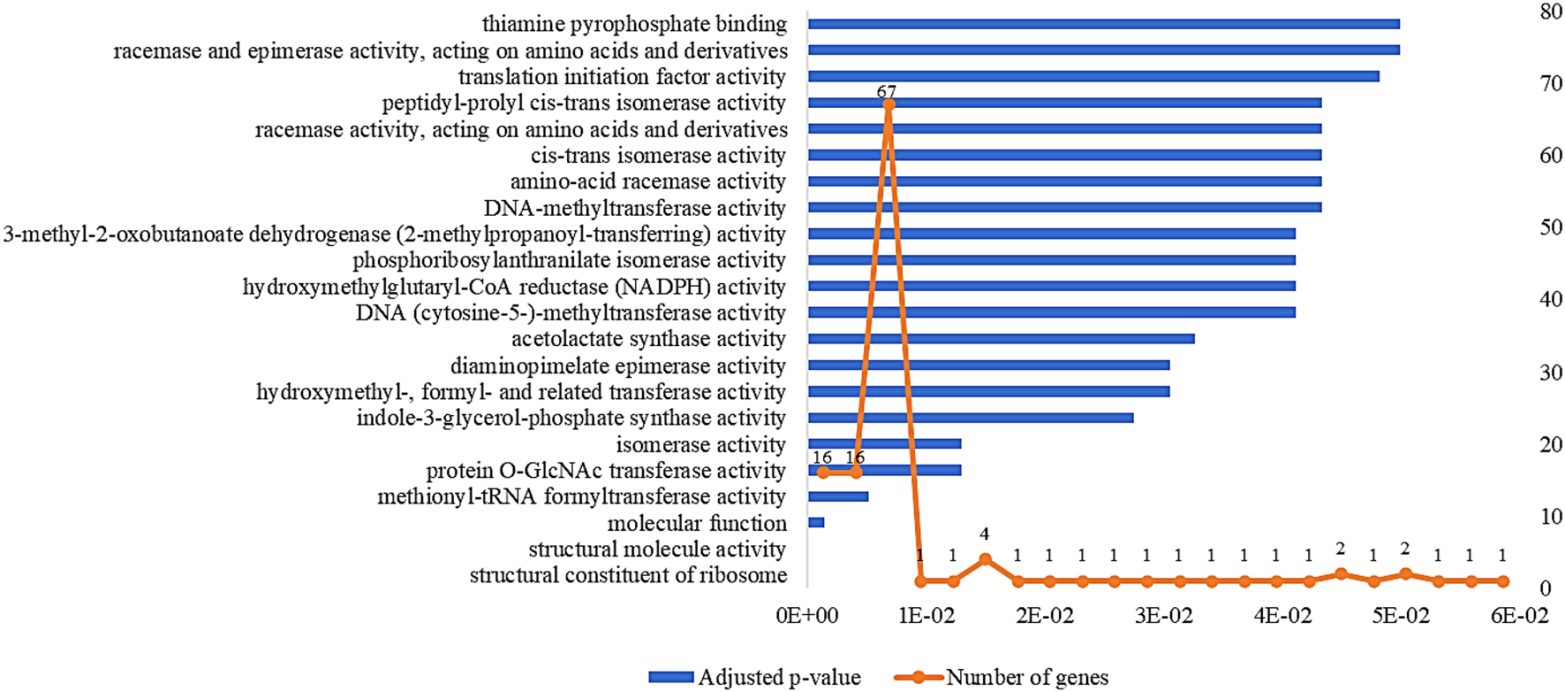
Molecular processes potentially involved in *B. megaterium*—tomato interaction

The outcome showed that from the 96 tomato genes included in the survey, 67 were classified into 22 GO molecular functions as shown in Fig. 3.

According to the results presented in Fig. 3, the molecular functions involved in *B. megaterium*–tomato interaction comprised pathways that play an important role in plant growth and development such as indole-3-glycerol-phosphate synthase activity, methionyl-tRNA formyltransferase, diaminopimelate epimerase activity, acetolactate synthase and translation initiation factor activity, due to having a role in the nitrogen compound metabolic process. The protein O-GlcNAc transferase activity pathway is correlated with gibberellins-mediated signaling pathway. Gibberellins are plant hormones that regulate various developmental processes (Binenbaum et al. 2018).

#### KEGG pathways

The outcome showed that from the 96 tomato genes included in the survey, 28 were classified into 20 KEGG pathways, as shown in Table 4.

**Table 4:** KEGG pathways for the target 96 tomato genes with Benjamini–Hochberg FDR < 0.05

Biological pathway	KEGG id	Adjusted p-value	Gene symbol
Ribosome	03010	1.96E–11	Solyc06g009210.3, Solyc01g103800.3, Solyc08g016180.3, Solyc09g075430.3, Solyc08g075700.3, Solyc02g070310.3 Solyc10g078960.2, Solyc06g008260.3 Solyc09g005720.3, Solyc09g075290.3 Solyc07g063910.3
KEGG root term	00000	5.61E–10	Solyc02g091290.3, Solyc06g009210.3 Solyc08g008210.3, Solyc09g010180.3 Solyc03g005050.3, Solyc12g006870.2 Solyc06g060720.3, Solyc01g103800.3 Solyc01g010020.3, Solyc02g082260.3 Solyc03g111850.3, Solyc07g006070.1 Solyc08g016180.3, Solyc08g078820.3 Solyc09g075430.3, Solyc01g110530.3 Solyc09g005700.3, Solyc08g075700.3 Solyc04g024530.3, Solyc02g070310.3 Solyc10g078960.2, Solyc06g008260.3 Solyc09g005720.3, Solyc10g083610.2 Solyc09g075290.3, Solyc07g063910.3 Solyc06g059840.3, Solyc08g067410.2
Metabolic pathways	01100	1.34E–02	Solyc02g091290.3, Solyc08g008210.3 Solyc03g005050.3, Solyc02g082260.3 Solyc03g111850.3, Solyc07g006070.1
One carbon pool by folate	00670	1.34E–02	Solyc02g091290.3
Other types of O-glycan biosynthesis	00514	1.38E–02	Solyc09g010180.3
Aminoacyl-tRNA biosynthesis	00970	1.56E–02	Solyc02g091290.3
D-Amino acid metabolism	00470	2.03E–02	Solyc09g005700.3
Thiamine metabolism	00730	2.96E–02	Solyc03g005050.3
Lysine biosynthesis	00300	3.14E–02	Solyc09g005700.3
Biosynthesis of secondary metabolites	01110	4.23E–02	Solyc03g005050.3, Solyc02g082260.3 Solyc03g111850.3, Solyc09g005700.3 Solyc06g059840.3, Solyc08g067410.2
Biosynthesis of amino acids	01230	4.25E–02	Solyc03g111850.3, Solyc09g005700.3
Purine metabolism	00230	4.41E–02	Solyc03g005050.3
Autophagy—other	04136	4.41E–02	Solyc08g078820.3
Viral life cycle—HIV-1	03250	4.41E–02	Solyc01g110530.3
Nucleotide metabolism	01232	4.41E–02	Solyc03g005050.3
Phagosome	04145	4.41E–02	Solyc08g008210.3
Oxidative phosphorylation	00190	4.58E–02	Solyc08g008210.3
Peroxisome	04146	4.78E–02	Solyc06g060720.3
Terpenoid backbone biosynthesis	00900	4.91E–02	Solyc02g082260.3
Phenylalanine, tyrosine and tryptophan biosynthesis	00400	4.91E–02	Solyc03g111850.3

KEGG enrichment results showed twenty pathways. The ‘Ribosome’ pathway belongs to the genetic information processing class. ‘Lysine biosynthesis’, ‘phenylalanine, tyrosine and tryptophan biosynthesis’ and ‘other types of O-glycan biosynthesis’ belong to the metabolism class. The lysine biosynthesis is a precursor for glutamate, an important signaling amino acid that regulates plant growth and responses to the environment (Galili 2002). The ‘phenylalanine, tyrosine and tryptophan biosynthesis’ pathway is involved in *B. megaterium*–tomato interaction. These aromatic amino acids in plants are not only essential components of protein synthesis, but also serve as precursors for a wide range of secondary metabolites that are important for plant growth (Tzin and Galili 2010). The ‘Peroxisome’ and the ‘autophagy-other’ pathways belong to the cellular processes class. Peroxisomes play vital roles in plant development, growth and environmental stress response (Su et al. 2019). ‘Biosynthesis of secondary metabolites’, ‘biosynthesis of amino acids’ and ‘metabolic pathways’ do not have associated classes. As indicated a recent work (Bhattacharya 2019) plant secondary metabolites play a significant role in plant adaptation to various environmental constraints that not only influence plant growth but also alter the biosynthesis of secondary metabolites. The ‘Thiamine metabolism’ and the ‘one carbon pool by folate’ pathways belong to the metabolism of cofactors and vitamins class. Thiamine plays an important role in plant’s primary regulatory system (Bocobza and Aharoni 2014) as an essential regulator (Subki et al. 2018). The ‘Phagosome’ belongs to cellular processes, transport and catabolism classes. The ‘Oxidative phosphorylation’ belongs to energy metabolism class. The ‘Purine metabolism’ pathway belongs to the nucleotide metabolism class and the ‘aminoacyl-tRNA biosynthesis’ pathway belongs to genetic information processing and translation classes.

### Biological networks analysis

**Fig. 4:**
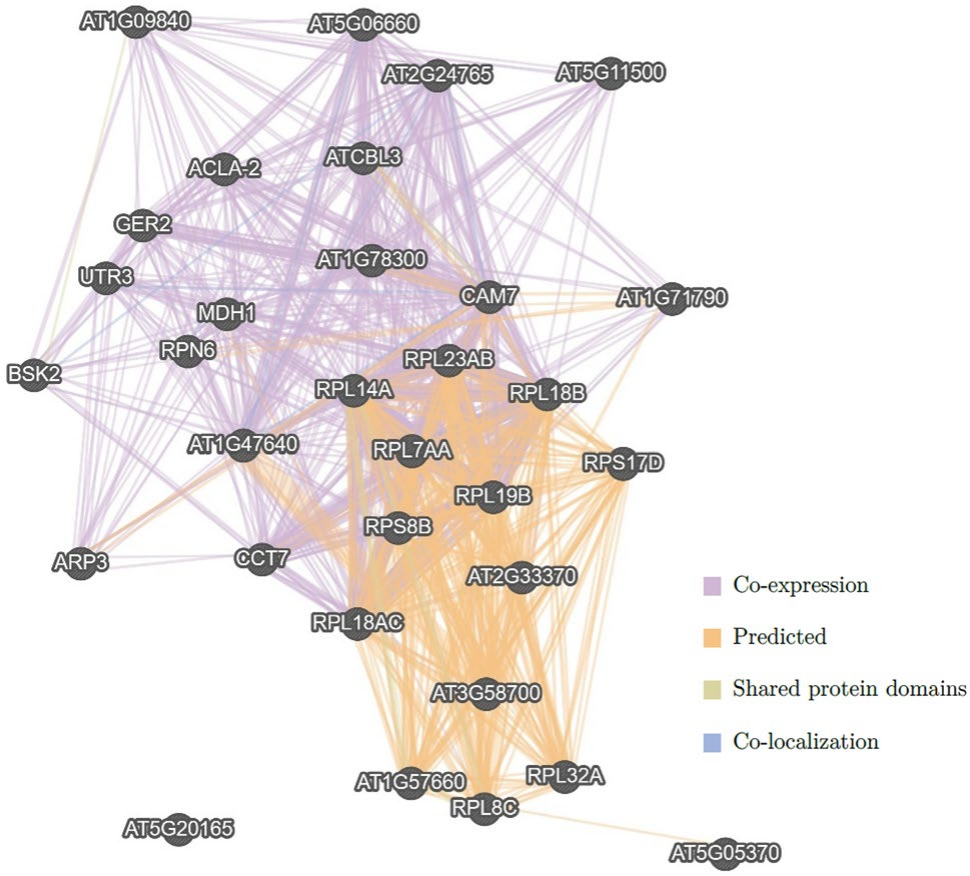
Gene interaction networks of the 32 *A. thaliana* orthologous genes in GeneMANIA Cytoscape database

Different networks of the 32 *A. thaliana* orthologous genes were composed to identify the possible biological networks involved in the 113 tomato genes. Applying the GO biological process based on network weighting, the outcome showed four interaction networks out of six possible, as shown in Fig. 4. There are presented co-expression data (38.94%), predicted interaction (16.20%), shared protein domains (3.67%), and co-localization data (0.33%) networks, producing a total of 2043 links.

According to meta-learning approach results, the bacterium identified is *B. subtilis*. We assume that due to *B. subtilis* is a well-studied bacterium, used as a model organism and because it has homologs genes, the probe ID for bacillus was appointed as *B. subtilis*. *E. coli* was also identified as producing biotin synthase. Biotin synthase is a member of the 'radical SAM' (S-adenosylmethionine) family, which converts DTB (dethiobiotin) into biotin (Lotierzo et al. 2005). As it is known ethylene plays a role in plant development, and it is biosynthesized from S-adenosylmethionine (Yang and Hoffman 1984). In *B. megaterium*–tomato interaction, it was also identified CTR1 (ethylene-inducible CTR1-like protein kinase) gene. This gene is involved in several biological processes, some of them are known for their important role in plant growth and development such as ethylene-mediated signaling pathway and gibberellin biosynthetic process.

## Conclusion

Optimally parameterized ML classifiers (SVM-a, SVM-b, KLR-a, SVM-b) and meta-learning algorithms of attribute-selected classifiers (meta-SVM-a, meta-SVM-b, meta-KLR-a, meta-KLR-b) were developed to predict *B. megaterium* in tomato root tissue. Among these, meta-KLR-b achieved near-optimal performance evaluation in the transcriptomic data. Subsequently, this meta learner identified 157 IGs of *B. megaterium*–tomato interaction: 113 tomato genes, 5 *B. subtilis* proteins, 1 *E. coli* protein and 6 attributes were unidentified.

Additionally, the dataset revealed 36 significantly differentially expressed genes (DEGs), and it was verified that among the IGs all DEGs were present.

The implementation of meta-learning approach not only identified the plant-bacteria genes interaction but also revealed the microorganisms that played a role at the time of sample extraction. Furthermore, from the 175 biological processes and 22 molecular functions identified in this interaction, most of them are known for their important role in plant development and growth. Regarding KEGG pathways were identified twenty pathways, for instance, biosynthesis of secondary metabolites, peroxisome, aminoacyl-tRNA biosynthesis and lysine biosynthesis. The biological networks obtained by application of *A. thaliana* orthologous genes showed 38.94% co-expression, 16.20% of predicted interaction, 3.67% of shared protein domains and 0.33% of co-localization, producing a total of 2043 links.

To our knowledge, this is the first analysis of *B. megaterium*– tomato root tissue interaction using meta-learning approach. Our results provide a novel method to identify potential plant-growth-promoting rhizobacteria and identify bacteria that have the same biological processes and molecular functions as *B. megaterium*. Although we have employed a novel methodology to analyze *B. megaterium*–tomato roots tissue interaction, the results showed consistency with previous approaches based on established methodologies.

## Supplementary Information

Below is the link to the electronic supplementary material.

Supplementary file1 (DOCX 64 KB)

## Data Availability

The lists of tomato genes identified through meta-KLR-b with a score of 1 and the biological processes for the 96 tomato genes are available in Appendix (Supplementary material). The data supporting the findings of this study are available from the corresponding author, (Sérgio Deusdado) upon request.
