# Supplementary material for: Meta-learning approach for bacteria classification and identification of informative genes of the Bacillus megaterium: tomato roots tissue interaction
Source: 3 Biotech. 2023 Jul 11;13(8):271. doi: 10.1007/s13205-023-03690-0 (PMC10335980; doi:10.1007/s13205-023-03690-0)
Supplement: Supplementary file 1 — Supplementary file1 (DOCX 64 KB) [file 13205_2023_3690_MOESM1_ESM.docx]

**Appendix A.** 113 Solanum lycopersicum genes identified through meta-KLR-b with a score of 1.

| **Probe ID** | **Gene ID** | **Description** |
| --- | --- | --- |
| LesAffx.13428.1.S1_at | LOC101258809 | uncharacterized LOC101258809 |
| Les.2878.1.S1_at | LOC101253694 | protein kish |
| Les.1338.1.A1_at | LOC101264494 | tRNA-specific adenosine deaminase TAD3 |
| Les.5785.1.S1_at | eIF(iso)4E | eukaryotic translation initiation factor iso4E |
| LesAffx.49844.1.S1_at | LOC101261082 | methionyl-tRNA formyltransferase |
| LesAffx.65023.1.S1_at | LOC101246078 | transmembrane emp24 domain-containing protein p24delta9 |
| LesAffx.39915.1.S1_at | LOC101267298 | transmembrane protein 147 |
| Les.4377.2.S1_at | LOC101261236 | 60S ribosomal protein L19-3 |
| LesAffx.67899.1.S1_at | LOC101254389 | N-acetyltransferase 9-like protein |
| LesAffx.109.1.A1_at | LOC101252546 | IDD1 C2H2-like zinc finger protein |
| Les.1221.2.S1_at | LOC101246256 | 60S ribosomal protein L7a-2 |
| Les.652.1.S1_at | LOC101247034 | T-complex protein 1 subunit eta |
| Les.795.2.S1_at | LOC101258750 | 26S proteasome non-ATPase regulatory subunit 11 homolog |
| Les.2219.1.A1_at | LOC101255735 | COP1-interacting protein 7 |
| LesAffx.68578.1.S1_at | LOC543663 | V-type proton ATPase subunit E |
| Les.3463.1.S1_at | spy | SPY protein |
| LesAffx.61214.1.S1_at | LOC101254065 | mitochondrial outer membrane protein porin of 34 kDa |
| LesAffx.2447.1.S1_at | LOC101259584 | 60S acidic ribosomal protein P1 |
| Les.2487.1.A1_at | LOC101251509 | serine/threonine-protein kinase BSK2 |
| Les.1978.2.S1_at | LOC101251751 | nucleoside/nucleotide kinase superfamily protein |
| Les.3022.2.S1_at | LOC101246764 | 60S ribosomal protein L11-1 |
| Les.463.1.S1_at | LOC101253806 | uncharacterized LOC101253806 |
| Les.1277.1.S1_at | LOC101268045 | mitochondrial import receptor subunit TOM20 |
| LesAffx.54871.1.S1_at | LOC101259414 | NADH dehydrogenase [ubiquinone] 1 beta subcomplex subunit 10-A |
| LesAffx.64252.1.S1_at | LOC101243765 | acyl-protein thioesterase 2 |
| Les.4229.1.S1_at | LOC101252997 | peroxisome biogenesis protein 19-2 |
| LesAffx.70507.1.S1_at | LOC101253835 | uncharacterized LOC101253835 |
| LesAffx.69816.1.S1_at | LOC101250979 | calcineurin subunit B |
| Les.3251.2.S1_at | LOC101247070 | 40S ribosomal protein S12 |
| Les.628.2.S1_at | LOC101259874 | S26 Type I signal peptidase family protein |
| Les.4305.1.S1_at | ureF | ureF protein |
| Les.3534.1.S1_at | LOC101268679 | cytochrome P450 81E8 |
| LesAffx.20507.1.S1_at | LOC101258334 | CASP-like protein 2D1 |
| LesAffx.57454.1.S1_at | LOC101262447 | probable calcium-binding protein CML15 |
| Les.94.1.S1_at | MET1 | DNA (cytosine-5)-methyltransferase 1 |
| LesAffx.70011.1.S1_at | LOC101266495 | uncharacterized LOC101266495 |
| Les.4844.1.S1_at | NA | NA |
| LesAffx.69699.1.S1_at | LOC101253307 | peptidyl-prolyl cis-trans isomerase CYP23 |
| Les.4372.1.S1_at | HMGR | 3-hydroxy-3-methylglutaryl-coenzyme A reductase 1 |
| LesAffx.66838.1.S1_at | LOC101249611 | calcineurin B-like protein |
| LesAffx.28368.1.S1_at | LOC101250390 | indole-3-glycerol phosphate synthase, chloroplastic |
| LesAffx.64732.1.S1_at | LOC101253202 | putative GDP-L-fucose synthase 2 |
| Les.2770.1.S1_at | LOC104644772 | uncharacterized LOC104644772 |
| Les.1064.1.S1_at | ALC | transcription factor ALC |
| Les.793.1.S1_s_at | LOC104648652 | 60S ribosomal protein L18a |
| LesAffx.67432.1.S1_at | LOC101266344 | ADP-ribosylation factor 1 |
| Les.2645.2.S1_at | CaM1 | calmodulin 1 |
| LesAffx.66052.1.S1_at | LOC101267820 | cytochrome b-c1 complex subunit 8 |
| Les.2513.1.S1_at | LOC100736469 | autophagy 8f |
| Les.1661.1.A1_at | LOC101248612 | translocase of chloroplast 159, chloroplastic |
| Les.895.1.A1_at | LOC101255132 | universal stress protein A-like protein |
| LesAffx.41758.1.S1_at | LOC101248263 | CBL-interacting serine/threonine-protein kinase 12-like |
| Les.2920.2.S1_at | LOC101268350 | elongation factor 1-beta 2 |
| Les.2496.2.S1_at | LOC101265769 | 60S ribosomal protein L19-3 |
| Les.2674.1.S1_at | LOC101267099 | probable NADH dehydrogenase [ubiquinone] 1 alpha subcomplex subunit 12 |
| Les.2636.2.S1_at | LOC101245005 | peptidyl-prolyl cis-trans isomerase Pin1 |
| Les.3272.1.S1_at | LOC101246955 | diaminopimelate epimerase, chloroplastic |
| Les.4012.1.S1_at | LOC101264241 | 60S ribosomal protein L13-1 |
| Les.4507.1.S1_at | LOC101252341 | 40S ribosomal protein S17 |
| Les.828.1.A1_at | LOC101255806 | probable inactive purple acid phosphatase 27 |
| LesAffx.3110.2.S1_at | LOC101256740 | dihydroceramide fatty acyl 2-hydroxylase FAH1 |
| Les.2881.2.S1_at | LOC101263520 | 60S ribosomal protein L23 |
| LesAffx.25974.1.A1_at | LOC101248208 | uncharacterized LOC101248208 |
| Les.4758.1.S1_at | LOC101252768 | actin-related protein 3 |
| Les.3378.2.S1_at | LOC101253131 | malate dehydrogenase |
| Les.5407.1.S1_at | LOC101246150 | probable serine/threonine protein kinase IREH1 |
| Les.2773.1.S1_at | LOC101253152 | 60S ribosomal protein L32-1 |
| LesAffx.179.1.S1_at | LOC101258695 | UPF0613 protein PB24D3.06c |
| Les.3365.1.S1_at | LOC101245989 | zinc finger CCCH domain-containing protein 30-like |
| LesAffx.68153.1.S1_at | LOC101256266 | probable F-actin-capping protein subunit beta |
| Les.4002.2.S1_at | LOC101257121 | 60S ribosomal protein L21-2 |
| LesAffx.46856.1.S1_at | LOC101260116 | heat shock factor-binding protein |
| LesAffx.56.3.S1_at | LOC101257776 | heat stress transcription factor A-6b |
| LesAffx.67341.1.S1_at | LOC101244542 | probable Histone-lysine N-methyltransferase ATXR5 |
| Les.3264.3.S1_at | LOC101261115 | sorting and assembly machinery component 50 homolog B |
| Les.3202.1.S1_at | LOC101254603 | 40S ribosomal protein S8 |
| LesAffx.51224.2.S1_at | LOC101259179 | coiled-coil domain-containing protein 25 |
| LesAffx.71372.1.S1_at | LOC101264160 | uncharacterized LOC101264160 |
| Les.5631.1.S1_at | LOC101258705 | uncharacterized LOC101258705 |
| Les.4754.1.S1_at | LOC101253061 | pectin acetylesterase 9 |
| LesAffx.71551.2.A1_at | LOC101264237 | aspartyl protease family protein At5g10770 |
| Les.292.1.S1_at | LOC101251606 | B2 protein |
| Les.1542.1.A1_at | LOC101254844 | uncharacterized protein At1g10890 |
| LesAffx.55662.1.S1_at | N/A | Thioredoxin domain-containing protein |
| Les.3036.2.S1_at | LOC101257715 | calcium load-activated calcium channel |
| LesAffx.56167.1.S1_at | LOC101250402 | glutathione S-transferase U17 |
| LesAffx.68102.1.A1_at | LOC101055522 | Hop-interacting protein THI037 |
| LesAffx.6103.1.S1_at | LOC101244376 | peroxidase 3 |
| Les.4800.1.S1_at | LOC101262229 or | probable 6-phosphogluconolactonase 4, chloroplastic |
| Les.2316.2.A1_at | LOC101255510 | cellulose synthase-like protein G2 |
| Les.2719.2.S1_at | LOC104647572 | 60S ribosomal protein L35a-3 |
| Les.3444.1.S1_at | LOC101248782 | uncharacterized LOC101248782 |
| Les.4613.1.S1_at | LOC101265073 | shaggy-related protein kinase kappa |
| Les.5188.1.S1_at | LOC101246414 | FRIGIDA-like protein 3 |
| Les.5772.1.S1_at | LOC101259686 | NADH dehydrogenase [ubiquinone] 1 alpha subcomplex subunit 1 |
| LesAffx.10198.1.A1_at | LOC101263792 | nucleoside-triphosphatase THEP1 |
| Les.3097.2.S1_at | LOC101265930 | 60S ribosomal protein L14-1 |
| LesAffx.3488.1.S1_at | LOC101248141 | malate dehydrogenase, chloroplastic |
| Les.4086.1.S1_at | LOC101246658 | 60S ribosomal protein L23a |
| Les.205.1.S1_at | LOC543565 | 14-3-3 family protein |
| Les.31.1.S1_s_at | CTR1 | ethylene-inducible CTR1-like protein kinase |
| Les.5063.1.S1_at | LOC101264913 | uncharacterized LOC101264913 |
| Les.4610.1.S1_at | LOC101267842 | 60S ribosomal protein L18-2 |
| LesAffx.32865.1.A1_at | LOC101257104 | peptidyl-prolyl cis-trans isomerase CYP21-4 |
| LesAffx.24212.1.A1_at | N/A | Uncharacterized protein |
| LesAffx.67767.1.A1_at | LOC101256601 | uncharacterized LOC101256601 |
| Les.3050.1.S1_at | RPL8 | ribosomal protein L2 |
| Les.5771.1.S1_at | LOC101247060 | SMAX1-LIKE protein |
| Les.3637.1.S1_at | LOC778303 | branched chain alpha-keto acid dehydrogenase E1-alpha subunit |
| LesAffx.17169.1.A1_at | LOC101246664 | K(+) efflux antiporter 2, chloroplastic |
| Les.3829.1.S1_at | LOC101261183 | pre-mRNA-splicing factor prp12 |
| Les.4040.1.S1_at | LOC101244691 | 3-ketoacyl-CoA synthase 11-like |
| Les.1389.1.S1_at | LE16 | non-specific lipid-transfer protein 2 |

**Appendix B.** 175 biological processes for the 96 tomato genes included in the survey.

| **Biological process** | **GO_id** | **Adjusted p-value** | **Number of genes** |
| --- | --- | --- | --- |
| peptide biosynthetic process | 43043 | 7.41E-12 | 18 |
| translation | 6412 | 7.41E-12 | 18 |
| peptide metabolic process | 6518 | 7.41E-12 | 19 |
| amide biosynthetic process | 43604 | 1.52E-11 | 18 |
| cellular amide metabolic process | 43603 | 1.67E-11 | 19 |
| organonitrogen compound biosynthetic process | 1901566 | 7.32E-11 | 22 |
| cellular macromolecule biosynthetic process | 34645 | 1.34E-10 | 19 |
| cellular process | 9987 | 1.04E-08 | 59 |
| organonitrogen compound metabolic process | 1901564 | 1.94E-07 | 33 |
| biological_process | 8150 | 1.95E-07 | 63 |
| cellular protein metabolic process | 44267 | 7.39E-07 | 26 |
| protein metabolic process | 19538 | 3.22E-06 | 27 |
| cellular macromolecule metabolic process | 44260 | 9.00E-06 | 27 |
| biosynthetic process | 9058 | 3.40E-05 | 27 |
| organic substance biosynthetic process | 1901576 | 6.40E-05 | 26 |
| cellular biosynthetic process | 44249 | 1.24E-04 | 25 |
| nitrogen compound metabolic process | 6807 | 1.64E-04 | 37 |
| cellular metabolic process | 44237 | 2.79E-04 | 40 |
| cellular nitrogen compound biosynthetic process | 44271 | 2.89E-04 | 20 |
| primary metabolic process | 44238 | 4.33E-04 | 40 |
| cellular nitrogen compound metabolic process | 34641 | 4.33E-04 | 25 |
| metabolic process | 8152 | 5.90E-04 | 43 |
| protein transport | 15031 | 6.12E-04 | 6 |
| organic substance metabolic process | 71704 | 6.12E-04 | 41 |
| establishment of protein localization | 45184 | 6.36E-04 | 6 |
| macromolecule biosynthetic process | 9059 | 7.24E-04 | 19 |
| intracellular transport | 46907 | 7.68E-04 | 6 |
| establishment of localization in cell | 51649 | 8.74E-04 | 6 |
| regulation of response to stimulus | 48583 | 8.74E-04 | 3 |
| protein localization | 8104 | 1.01E-03 | 6 |
| negative regulation of defense response to virus | 50687 | 1.15E-03 | 1 |
| conversion of methionyl-tRNA to N-formyl-methionyl-tRNA | 71951 | 1.48E-03 | 1 |
| intracellular protein transport | 6886 | 1.63E-03 | 5 |
| negative regulation of response to stimulus | 48585 | 1.66E-03 | 2 |
| cellular localization | 51641 | 1.72E-03 | 6 |
| nitrogen compound transport | 71705 | 2.43E-03 | 6 |
| charged-tRNA amino acid modification | 19988 | 2.56E-03 | 1 |
| macromolecule localization | 33036 | 2.63E-03 | 6 |
| gene expression | 10467 | 2.63E-03 | 19 |
| regulation of defense response to virus | 50688 | 2.67E-03 | 1 |
| cellular protein localization | 34613 | 2.78E-03 | 5 |
| cellular macromolecule localization | 70727 | 2.78E-03 | 5 |
| negative regulation of response to external stimulus | 32102 | 3.24E-03 | 1 |
| negative regulation of response to biotic stimulus | 2832 | 3.24E-03 | 1 |
| calcium-mediated signaling | 19722 | 4.93E-03 | 2 |
| second-messenger-mediated signaling | 19932 | 5.17E-03 | 2 |
| organic substance transport | 71702 | 5.41E-03 | 6 |
| regulation of signaling | 23051 | 5.56E-03 | 2 |
| regulation of signal transduction | 9966 | 5.56E-03 | 2 |
| regulation of cell communication | 10646 | 5.57E-03 | 2 |
| negative regulation of defense response | 31348 | 5.58E-03 | 1 |
| protein secretion | 9306 | 5.71E-03 | 1 |
| establishment of protein localization to extracellular region | 35592 | 5.71E-03 | 1 |
| protein localization to extracellular region | 71692 | 5.71E-03 | 1 |
| negative regulation of gibberellic acid mediated signaling pathway | 9938 | 6.47E-03 | 1 |
| cell communication | 7154 | 6.83E-03 | 7 |
| protein insertion into mitochondrial outer membrane | 45040 | 9.00E-03 | 1 |
| outer mitochondrial membrane organization | 7008 | 9.00E-03 | 1 |
| macromolecule metabolic process | 43170 | 1.05E-02 | 29 |
| C-5 methylation of cytosine | 90116 | 1.24E-02 | 1 |
| DNA methylation on cytosine within a CG sequence | 10424 | 1.24E-02 | 1 |
| defense response to symbiont | 140546 | 1.57E-02 | 1 |
| defense response to virus | 51607 | 1.57E-02 | 1 |
| signal transduction | 7165 | 1.57E-02 | 6 |
| regulation of reactive oxygen species metabolic process | 2000377 | 1.62E-02 | 1 |
| signaling | 23052 | 1.62E-02 | 6 |
| regulation of ARF protein signal transduction | 32012 | 1.63E-02 | 1 |
| ARF protein signal transduction | 32011 | 1.63E-02 | 1 |
| DNA methylation on cytosine | 32776 | 1.63E-02 | 1 |
| response to virus | 9615 | 1.63E-02 | 1 |
| transport | 6810 | 1.70E-02 | 8 |
| regulation of gibberellic acid mediated signaling pathway | 9937 | 1.73E-02 | 1 |
| regulation of small GTPase mediated signal transduction | 51056 | 1.73E-02 | 1 |
| regulation of Ras protein signal transduction | 46578 | 1.73E-02 | 1 |
| establishment of localization | 51234 | 1.74E-02 | 8 |
| regulation of response to biotic stimulus | 2831 | 1.78E-02 | 1 |
| regulation of response to external stimulus | 32101 | 1.80E-02 | 1 |
| cytoskeleton organization | 7010 | 1.81E-02 | 3 |
| gibberellic acid mediated signaling pathway | 9740 | 2.04E-02 | 1 |
| branched-chain amino acid metabolic process | 9081 | 2.04E-02 | 2 |
| localization | 51179 | 2.04E-02 | 8 |
| cellular response to gibberellin stimulus | 71370 | 2.04E-02 | 1 |
| endoplasmic reticulum to Golgi vesicle-mediated transport | 6888 | 2.04E-02 | 2 |
| translational initiation | 6413 | 2.04E-02 | 1 |
| gibberellin mediated signaling pathway | 10476 | 2.04E-02 | 1 |
| intracellular signal transduction | 35556 | 2.04E-02 | 4 |
| secretion by cell | 32940 | 2.04E-02 | 1 |
| secretion | 46903 | 2.07E-02 | 1 |
| endoplasmic reticulum calcium ion homeostasis | 32469 | 2.08E-02 | 1 |
| maintenance of DNA methylation | 10216 | 2.11E-02 | 1 |
| regulation of defense response | 31347 | 2.15E-02 | 1 |
| actin filament organization | 7015 | 2.31E-02 | 2 |
| regulation of protein depolymerization | 1901879 | 2.32E-02 | 1 |
| actin filament capping | 51693 | 2.32E-02 | 1 |
| spindle pole body organization | 51300 | 2.32E-02 | 1 |
| export from cell | 140352 | 2.32E-02 | 1 |
| negative regulation of protein depolymerization | 1901880 | 2.32E-02 | 1 |
| regulation of actin filament depolymerization | 30834 | 2.32E-02 | 1 |
| negative regulation of protein-containing complex disassembly | 43242 | 2.32E-02 | 1 |
| negative regulation of actin filament depolymerization | 30835 | 2.32E-02 | 1 |
| barbed-end actin filament capping | 51016 | 2.32E-02 | 1 |
| regulation of translation | 6417 | 2.41E-02 | 1 |
| actin filament-based process | 30029 | 2.41E-02 | 2 |
| actin cytoskeleton organization | 30036 | 2.41E-02 | 2 |
| dicarboxylic acid metabolic process | 43648 | 2.41E-02 | 2 |
| Golgi organization | 7030 | 2.41E-02 | 1 |
| regulation of cellular amide metabolic process | 34248 | 2.41E-02 | 1 |
| ion transmembrane transport | 34220 | 2.42E-02 | 2 |
| cellular response to stimulus | 51716 | 2.42E-02 | 7 |
| regulation of cellular macromolecule biosynthetic process | 2000112 | 2.55E-02 | 1 |
| organelle organization | 6996 | 2.58E-02 | 7 |
| cellular component organization or biogenesis | 71840 | 2.66E-02 | 10 |
| tRNA modification | 6400 | 2.68E-02 | 1 |
| cytokinin-activated signaling pathway | 9736 | 2.71E-02 | 1 |
| establishment of protein localization to mitochondrial membrane | 90151 | 2.71E-02 | 1 |
| vesicle-mediated transport | 16192 | 2.71E-02 | 3 |
| protein insertion into mitochondrial membrane | 51204 | 2.71E-02 | 1 |
| cellular response to cytokinin stimulus | 71368 | 2.71E-02 | 1 |
| regulation of response to stress | 80134 | 2.71E-02 | 1 |
| coenzyme A metabolic process | 15936 | 2.84E-02 | 1 |
| mitochondrial membrane organization | 7006 | 3.01E-02 | 1 |
| regulation of intracellular signal transduction | 1902531 | 3.14E-02 | 1 |
| endomembrane system organization | 10256 | 3.27E-02 | 1 |
| Golgi vesicle transport | 48193 | 3.36E-02 | 2 |
| post-transcriptional regulation of gene expression | 10608 | 3.41E-02 | 1 |
| supramolecular fiber organization | 97435 | 3.41E-02 | 2 |
| lysine biosynthetic process via diaminopimelate | 9089 | 3.42E-02 | 1 |
| diaminopimelate metabolic process | 46451 | 3.42E-02 | 1 |
| tRNA processing | 8033 | 3.48E-02 | 1 |
| negative regulation of signaling | 23057 | 3.49E-02 | 1 |
| negative regulation of cell communication | 10648 | 3.49E-02 | 1 |
| negative regulation of signal transduction | 9968 | 3.49E-02 | 1 |
| organic acid biosynthetic process | 16053 | 3.55E-02 | 4 |
| indolalkylamine biosynthetic process | 46219 | 3.56E-02 | 1 |
| tryptophan biosynthetic process | 162 | 3.56E-02 | 1 |
| branched-chain amino acid biosynthetic process | 9082 | 3.63E-02 | 1 |
| lysine metabolic process | 6553 | 3.63E-02 | 1 |
| lysine biosynthetic process | 9085 | 3.63E-02 | 1 |
| protein insertion into membrane | 51205 | 3.68E-02 | 1 |
| mitochondrial electron transport, ubiquinol to cytochrome c | 6122 | 3.68E-02 | 1 |
| response to gibberellin | 9739 | 3.68E-02 | 1 |
| microtubule organizing center organization | 31023 | 3.70E-02 | 1 |
| negative regulation of protein-containing complex assembly | 31333 | 3.70E-02 | 1 |
| negative regulation of actin filament polymerization | 30837 | 3.70E-02 | 1 |
| negative regulation of supramolecular fiber organization | 1902904 | 3.70E-02 | 1 |
| cellular amino acid metabolic process | 6520 | 3.70E-02 | 4 |
| carboxylic acid metabolic process | 19752 | 3.70E-02 | 6 |
| negative regulation of cytoskeleton organization | 51494 | 3.70E-02 | 1 |
| negative regulation of protein polymerization | 32272 | 3.70E-02 | 1 |
| DNA methylation | 6306 | 3.75E-02 | 1 |
| DNA alkylation | 6305 | 3.75E-02 | 1 |
| alpha-amino acid biosynthetic process | 1901607 | 3.77E-02 | 2 |
| cellular response to nitrogen starvation | 6995 | 3.77E-02 | 1 |
| protein targeting | 6605 | 3.77E-02 | 2 |
| Arp2/3 complex-mediated actin nucleation | 34314 | 3.77E-02 | 1 |
| protein targeting to chloroplast | 45036 | 3.78E-02 | 1 |
| protein localization to chloroplast | 72598 | 3.78E-02 | 1 |
| establishment of protein localization to chloroplast | 72596 | 3.78E-02 | 1 |
| oxoacid metabolic process | 43436 | 3.85E-02 | 6 |
| regulation of protein-containing complex disassembly | 43244 | 3.89E-02 | 1 |
| establishment of protein localization to organelle | 72594 | 3.90E-02 | 2 |
| small molecule metabolic process | 44281 | 3.96E-02 | 8 |
| actin nucleation | 45010 | 3.97E-02 | 1 |
| indole-containing compound biosynthetic process | 42435 | 3.99E-02 | 1 |
| negative regulation of biological process | 48519 | 4.03E-02 | 2 |
| organic acid metabolic process | 6082 | 4.24E-02 | 6 |
| tRNA metabolic process | 6399 | 4.24E-02 | 1 |
| ribosomal large subunit biogenesis | 42273 | 4.24E-02 | 2 |
| nucleotide-sugar biosynthetic process | 9226 | 4.50E-02 | 1 |
| carbohydrate derivative metabolic process | 1901135 | 4.54E-02 | 3 |
| DNA methylation or demethylation | 44728 | 4.60E-02 | 1 |
| ribosome biogenesis | 42254 | 4.60E-02 | 3 |
| cellular amino acid biosynthetic process | 8652 | 4.63E-02 | 2 |
| aerobic respiration | 9060 | 4.72E-02 | 2 |
| DNA modification | 6304 | 4.94E-02 | 1 |
